# Supplementary material for: Structural and functional analyses explain Pea KAI2 receptor diversity and reveal stereoselective catalysis during signal perception
Source: Commun Biol. 2022 Feb 11;5:126. doi: 10.1038/s42003-022-03085-6 (PMC8837635; doi:10.1038/s42003-022-03085-6)
Supplement: Supplementary file 1 — Supplementary Information [file 42003_2022_3085_MOESM1_ESM.pdf]

Structural and Functional Analyses Explain Pea KAI2 Receptor Diversity and Reveal Stereoselective Catalysis During Signal Perception

Angelica M. Guercio[1], Salar Torabi[2], David Cornu[3], Marion Dalmais[4], Abdelhafid Bendahmane[4], Christine Le Signor[5], Jean-Paul Pillot[6], Philippe Le Bris[6], François-Didier Boyer[7], Catherine Rameau[6], Caroline Gutjahr[2], Alexandre de Saint Germain[6]*, and Nitzan Shabek[1]*

Supplementary Materials

Supplementary Fig. 1. Proteins Sequence analysis of KAI2/D14 family.

Supplementary Fig. 2. Alternative splicing of the *PsKAI2A* transcript and expression analysis of *PsKAI2C* pseudogene.

Supplementary Fig. 3. Multiple sequence alignment and conservation analysis of representative legume and non-legume KAI2s.

Supplementary Fig. 4. TILLING mutant residues position on KAI2 structures.

Supplementary Fig. 5. Branching and root hair phenotypes *of Pskai2* mutants.

Supplementary Fig. 6. Hypocotyl elongation in Ler *kai2-2* mutant and KAI2s protein expression in complementation assay.

Supplementary Fig. 7. Purification of PsKAI2 Proteins.

Supplementary Fig. 8. Biochemical analysis of the interaction between PsKAI2 proteins and the (+)-2'-epi-GR24 and (-)-2'-epi-GR24 isomers ligands by DSF.

Supplementary Fig. 9. Intrinsic tryptophan fluorescence of PsKAI2s, AtKAI2 and RMS3 proteins in the presence of SL analogs.

Supplementary Fig. 10. Structural divergence analysis of legume KAI2A and KAI2B and effect on ligand docking.

Supplementary Fig. 11. Biochemical and structural analysis of the interaction between wildtype and residue 160 and 190 swap mutant PsKAI2 proteins and (–)-GR24 by DSF and effect on ligand docking.

Supplementary Fig. 12. Structural interrogation of the ligand bound PsKAI2B crystal structure.

Supplementary Fig. 13. Mass spectrometry characterization of covalent PsKAI2-ligand complexes.

Supplementary Table 1. List of the mutations identified during TILLING and mutant alleles used in the study.

Supplementary Table 2. Primer sequences used in study.

a

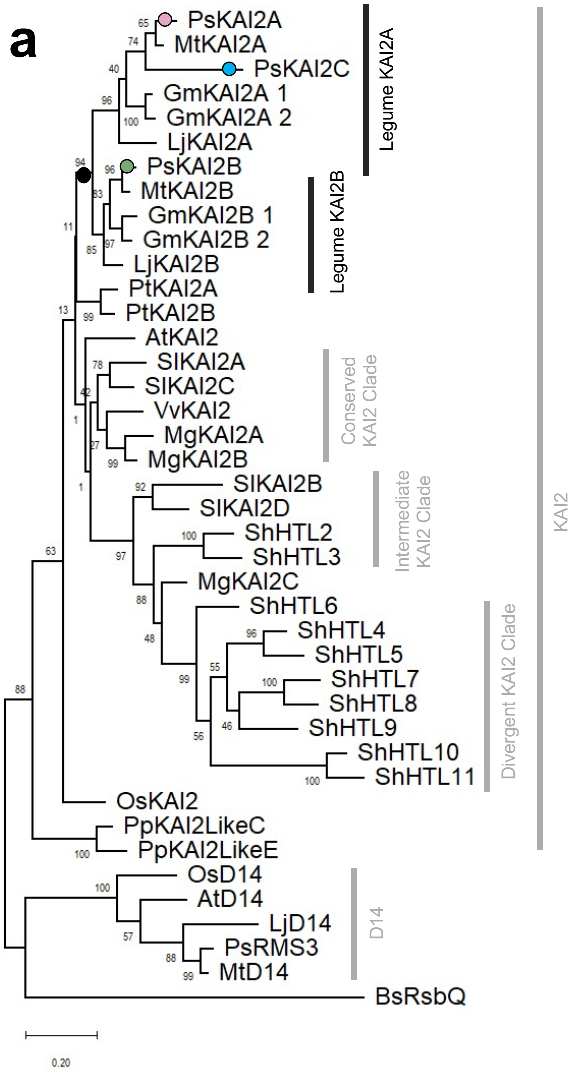

b

	Species	Gene name	Sequence ID	Source
	Pisum sativum	KAI2A	Psat2g169960	ANR GENOPEA
	Pisum sativum	KAI2B	Psat4g083040	ANR GENOPEA
	Pisum sativum	KAI2C	Psat3g014200	ANR GENOPEA
	Oryza sativa	KAI2	LOC_Os03g32270.1	Phytozome
	Arabidopsis thaliana	KAI2	AT4G37470.1	TAIR
	Populus trichocarpa	KAI2A	Potri.005G145000.1	Phytozome
	Populus trichocarpa	KAI2B	Potri.007G052000.1	Phytozome
	Lotus japonicus	KAI2A	Lj2g3v1931930.1	LORE1
	Lotus japonicus	KAI2B	Lj0g3v0117039.1	LORE1
	Solanum Lycopersicon	KAI2A	Solyc02g064770.2.1	Phytozome
	Solanum Lycopersicon	KAI2B	Solyc02g064760.1.1	Phytozome
	Solanum Lycopersicon	KAI2C	Solyc02g092770.2.1	Phytozome
	Solanum Lycopersicon	KAI2D	Solyc02g092760.2.1	Phytozome
	Vitis vinifera	KAI2	GSVIVT01000162001	Phytozome
	Mimulus guttatus	KAI2A	Migut.H00375.1	Phytozome
	Mimulus guttatus	KAI2B	Migut.H00378.1	Phytozome
	Mimulus guttatus	KAI2C	Migut.L00194.1	Phytozome
KAI2	Physcomitrella patens	KAI2-LikeC	6ATX	RCSB
	Physcomitrella patens	KAI2-LikeE	6AZB	RCSB
	Striga hermonthica	HTL2	from Toh et al 2015	Toh et al 2015[56]
	Striga hermonthica	HTL3	from Toh et al 2015	Toh et al 2015[56]
	Striga hermonthica	HTL4	from Toh et al 2015	Toh et al 2015[56]
	Striga hermonthica	HTL5	from Toh et al 2015	Toh et al 2015[56]
	Striga hermonthica	HTL6	from Toh et al 2015	Toh et al 2015[56]
	Striga hermonthica	HTL7	from Toh et al 2015	Toh et al 2015[56]
	Striga hermonthica	HTL8	from Toh et al 2015	Toh et al 2015[56]
	Striga hermonthica	HTL9	from Toh et al 2015	Toh et al 2015[56]
	Striga hermonthica	HTL10	from Toh et al 2015	Toh et al 2015[56]
	Striga hermonthica	HTL11	from Toh et al 2015	Toh et al 2015[56]
	Glycine max	KAI2A 1	Glyma.01G191200.1.p	Phytozome
	Glycine max	KAI2A 2	Glyma.11G051000.1.p	Phytozome
	Glycine max	KAI2B 1	Glyma.17G164500.1.p	Phytozome
	Glycine max	KAI2B 2	Glyma.05G102800.1.p	Phytozome
	Medicago truncatula	KAI2A	Medtr5g016150.1	Phytozome
	Medicago truncatula	KAI2B	Medtr4g095310.1	Phytozome
	Pisum sativum	RMS3	Psat6g018320	ANR GENOPEA
	Oryza sativa	D14	3W04	RCSB
D14	Arabidopsis thaliana	D14	AT3G03990	TAIR
	Lotus japonicus	D14	Lj5g3v0310140.4	LORE1
	Medicago truncatula	D14	Medtr1g018320.1	Phytozome
RsbQ	Bacillus subtilis	RsbQ	WP_187001895	NCBI

Supplementary Fig. 1. Proteins Sequence analysis of KAI2/D14 family. (**a**) Maximum likelihood phylogeny of 41 KAI2/D14-family proteins with bacterial RBSQ* as outgroup. Node values represent percentage of trees in which the associated taxa clustered together. Vertical rectangles highlight distinct *KAI2/D14* family clades. Black circle indicates legume duplication event. Pink and green circles mark the position of *PsKAI2As* and *PsKAI2B* respectively. The tree is drawn to scale, with branch lengths measured in the number of substitutions per site. (**b**) Sequence identifiers and sources for each. *RBSQ is a bacterial hydrolase with the most similar a/b fold.

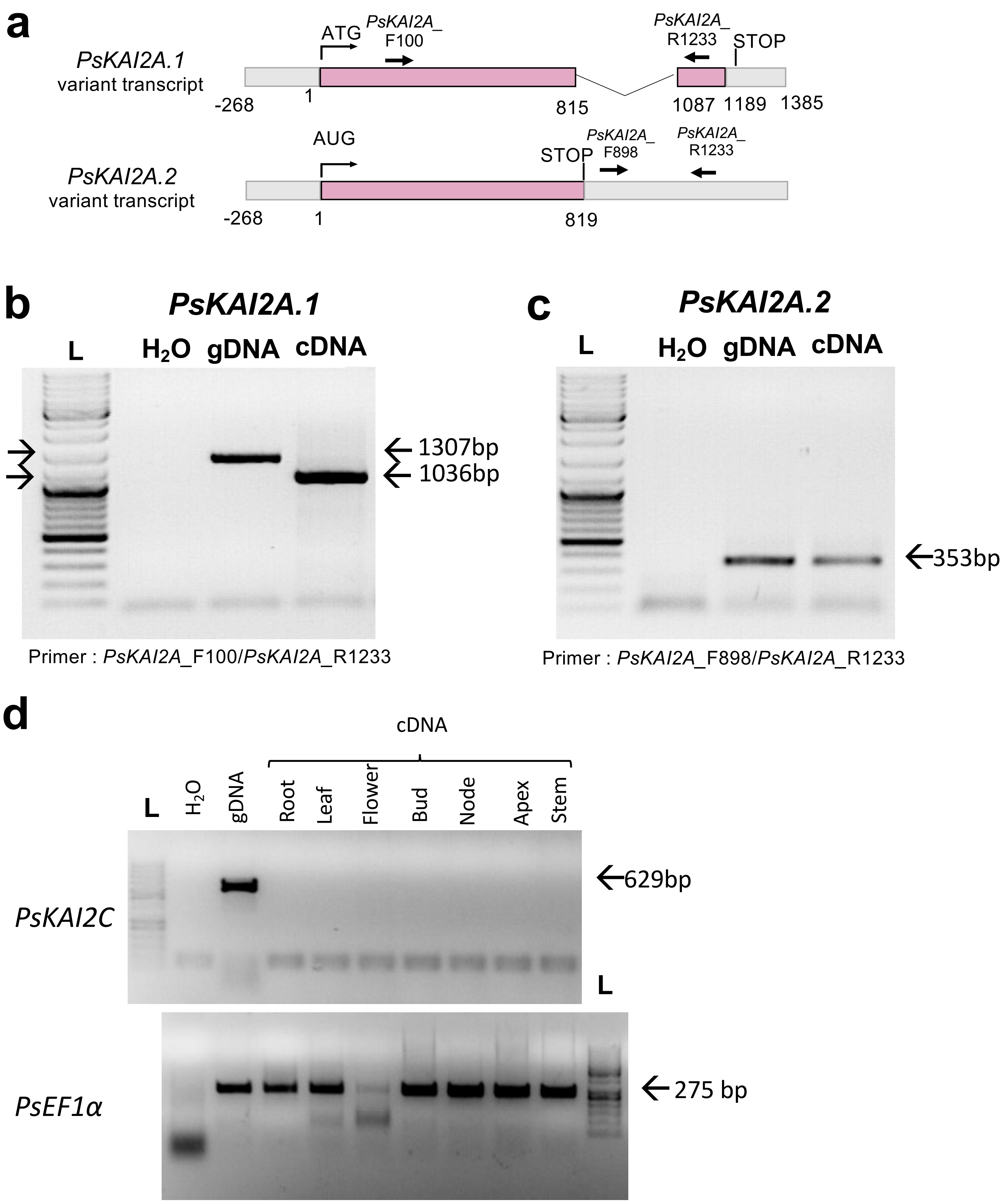

Supplementary Fig. 2. Alternative splicing of the *PsKAI2A* transcript and expression analysis of *PsKAI2C* pseudogene. (**a**) Schematic representation of the splicing events leading to *PsKAI2A.1* and *PsKAI2A.2* forms of mature transcripts. Exons are indicated as pink boxes. (**b-c**) Electrophoresis gel of PCR products obtained after amplification of the PsKAI2A coding sequence with primers specific to *PsKAI2A.1* form (**b**) and primers specific to *PsKAI2A.2* form (**c**) using genomic DNA (gDNA) or first-strand cDNA (cDNA) as template; H2O: negative control; Lane L: DNA ladder. (**d**) Electrophoresis gel of PCR products obtained after amplification of the predicted *PsKAI2C* coding sequence and the reference gene *PsEF1α* using genomic DNA (gDNA) or first-strand cDNA (cDNA) from different tissues as template; H2O: negative control; Lane L: DNA ladder.

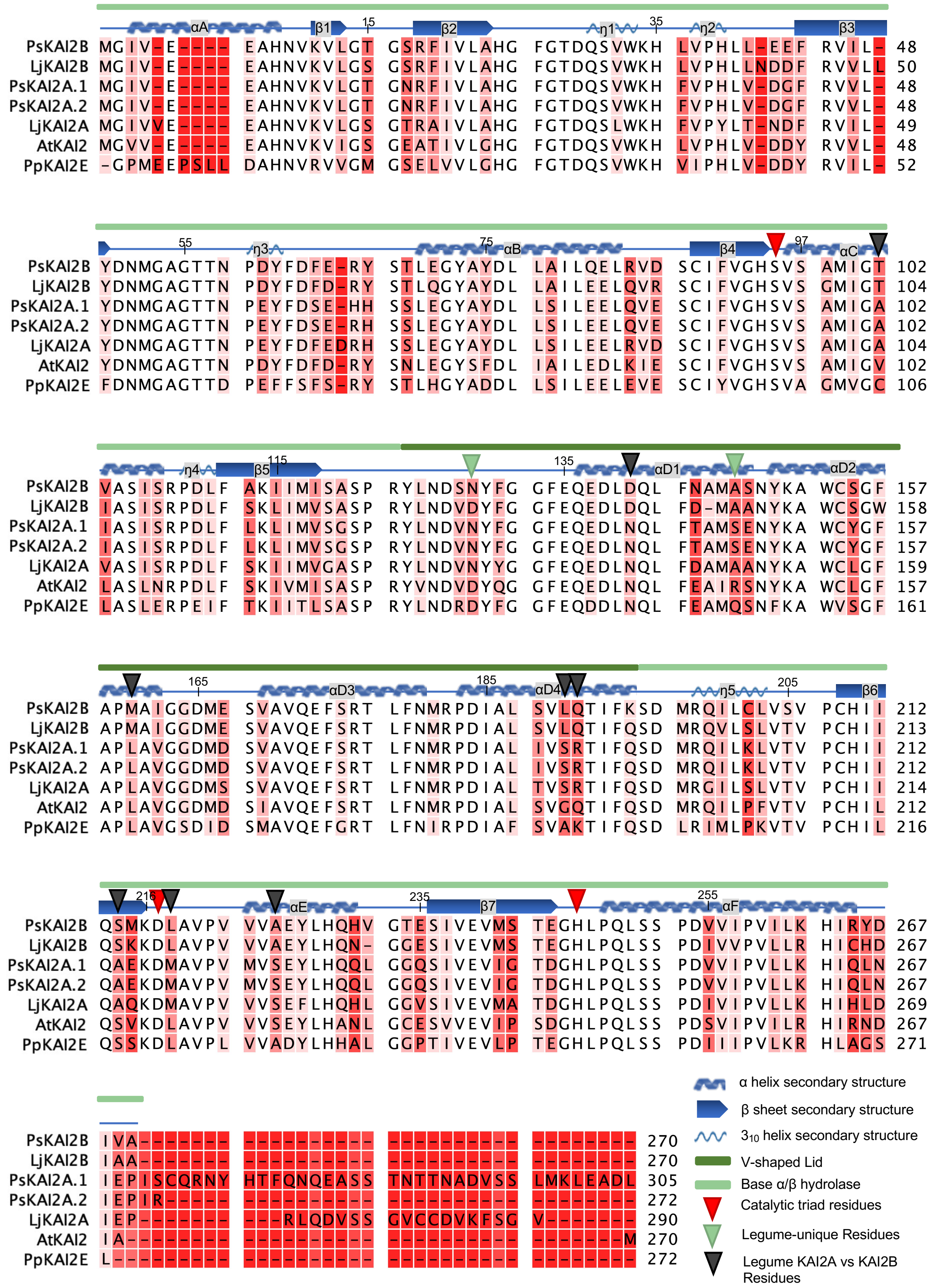

Supplementary Fig. 3. Multiple sequence alignment and conservation analysis of representative legume and non-legume KAI2s. Multiple sequence alignment and conservation analysis of selected KAI2s. Amino acid alignment of 7 plant KAI2 proteins. Intensity of red behind residues shows degree of divergence. Numbers on residues refer to position in PsKAI2B sequence. PsKAI2B lid (forest green) and base (light green) domains are indicated above alignment. Secondary structure of PsKAI2B sequence is shown above sequence in blue as alpha helices (αA- αF, base; and αD1- αD4, lid), beta sheets (β1- β7), 310 helices (η1- η5) and non-secondary structure-containing loops. Red arrows indicate catalytic triad residues, green arrows indicate legume KAI2-unique residues as shown in **Fig. 6**, dark grey arrows indicate residues diverged between legume KAI2A and KAI2B as described in **Fig. 7** and **Supplementary Fig. 10**.

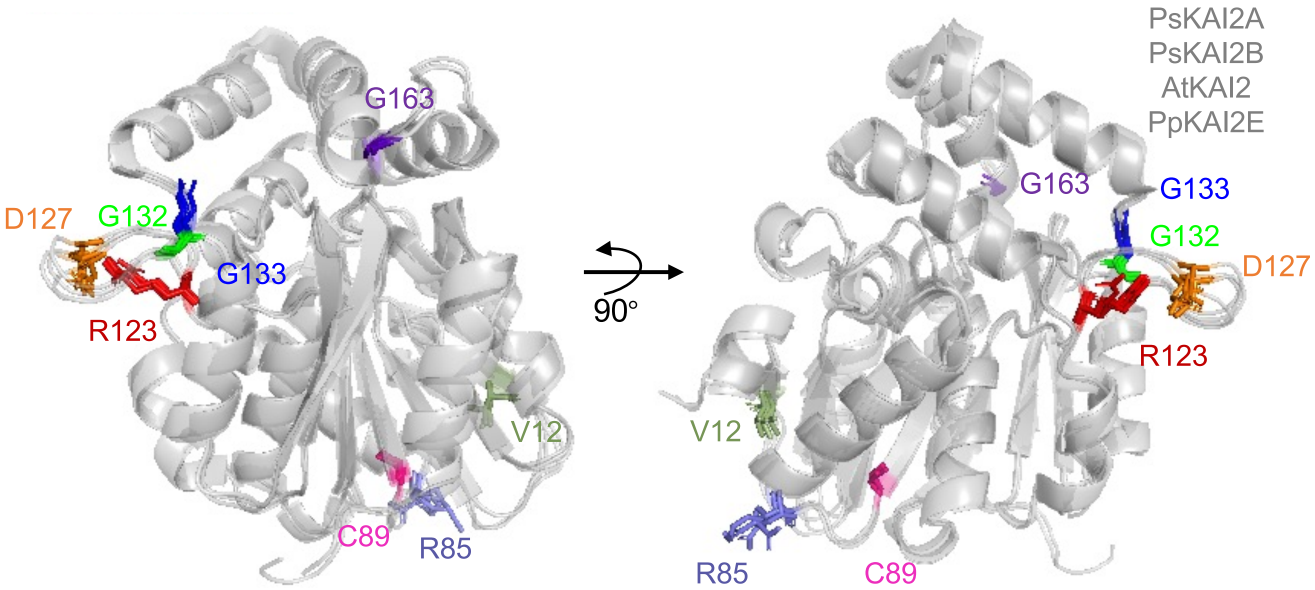

Supplementary Fig. 4. TILLING mutant residues position on KAI2 structures. Superposition of PsKAI2A, PsKAI2B, and example KAI2s AtKAI2 (PDB ID: 4HTA), and PpKAI2E (PDB ID: 6AZB) structures are shown in grey. The identified TILLING mutant residues from **Fig. 2a** are highlighted and shown as sticks and labelled in rainbow colors. PsKAI2B was used as the reference for amino acid sequence position.

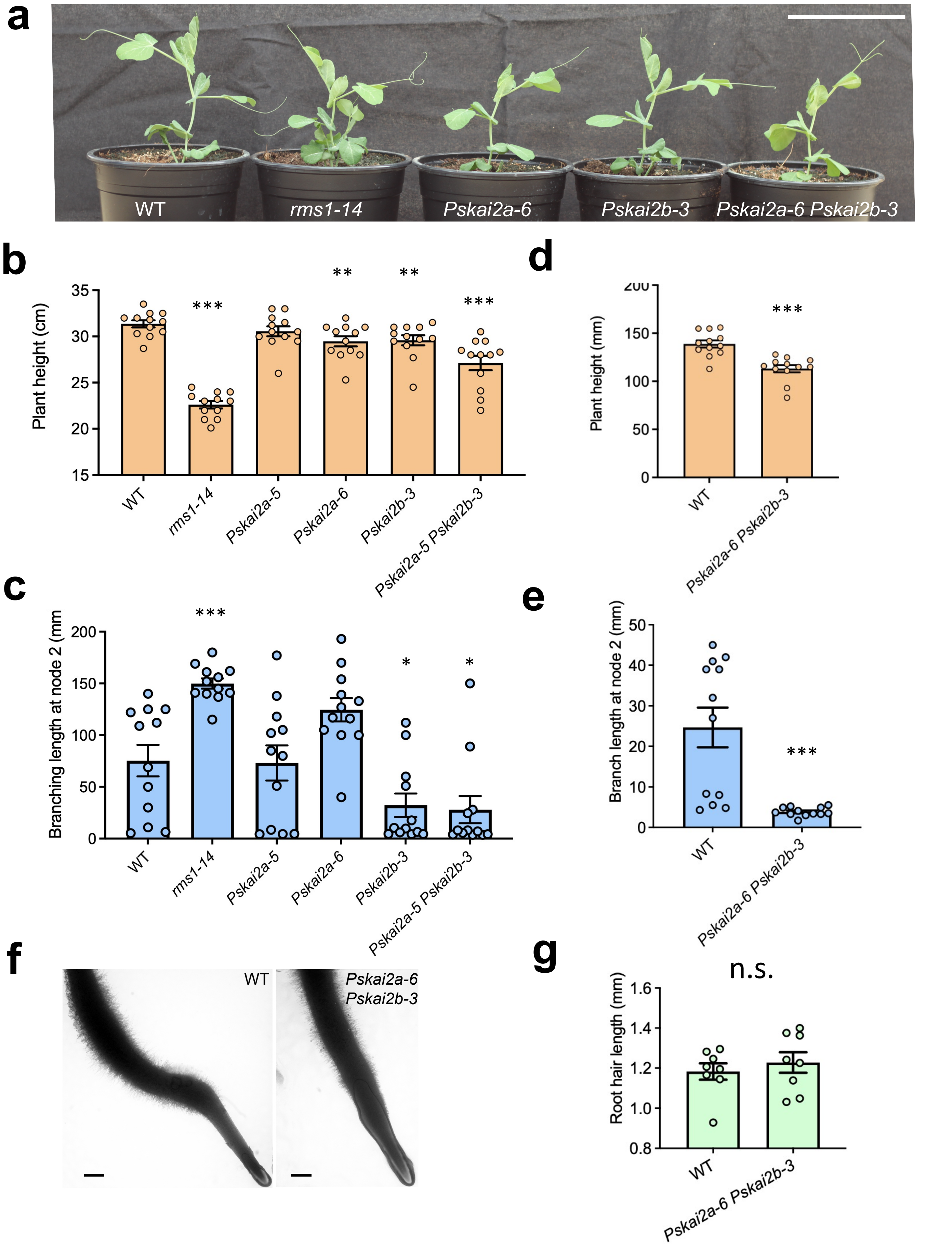

Supplementary Fig. 5. Branching and root hair phenotypes of *Pskai2* mutants. (**a**) Phenotype and quantification of (**b-d**) plant height and (**c-e**) branch length at node 2 (mm) of 20-day-old (**b-c**) or 10-day-old (**d-e**) *Pisum sativum* plant. Bar, 8.5 cm. Data are means ± SE (n=11-12). Statistical differences were determined using the Kruskal-Wallis rank sum test (Asterisks indicate significant differences between WT and the mutant *P < 0.05 , **P< 0.01 and ***P < 0.001). (**f**) Root hair length phenotype and quantification (**g**) of 10 day old WT and *Pskai2a-6 Pskai2b-3* seedlings. Bars, 2 mm. Data are means of measurements (n=8) of individual root (n=8-9), Statistical analysis Welch t-test, p-value < 0.05

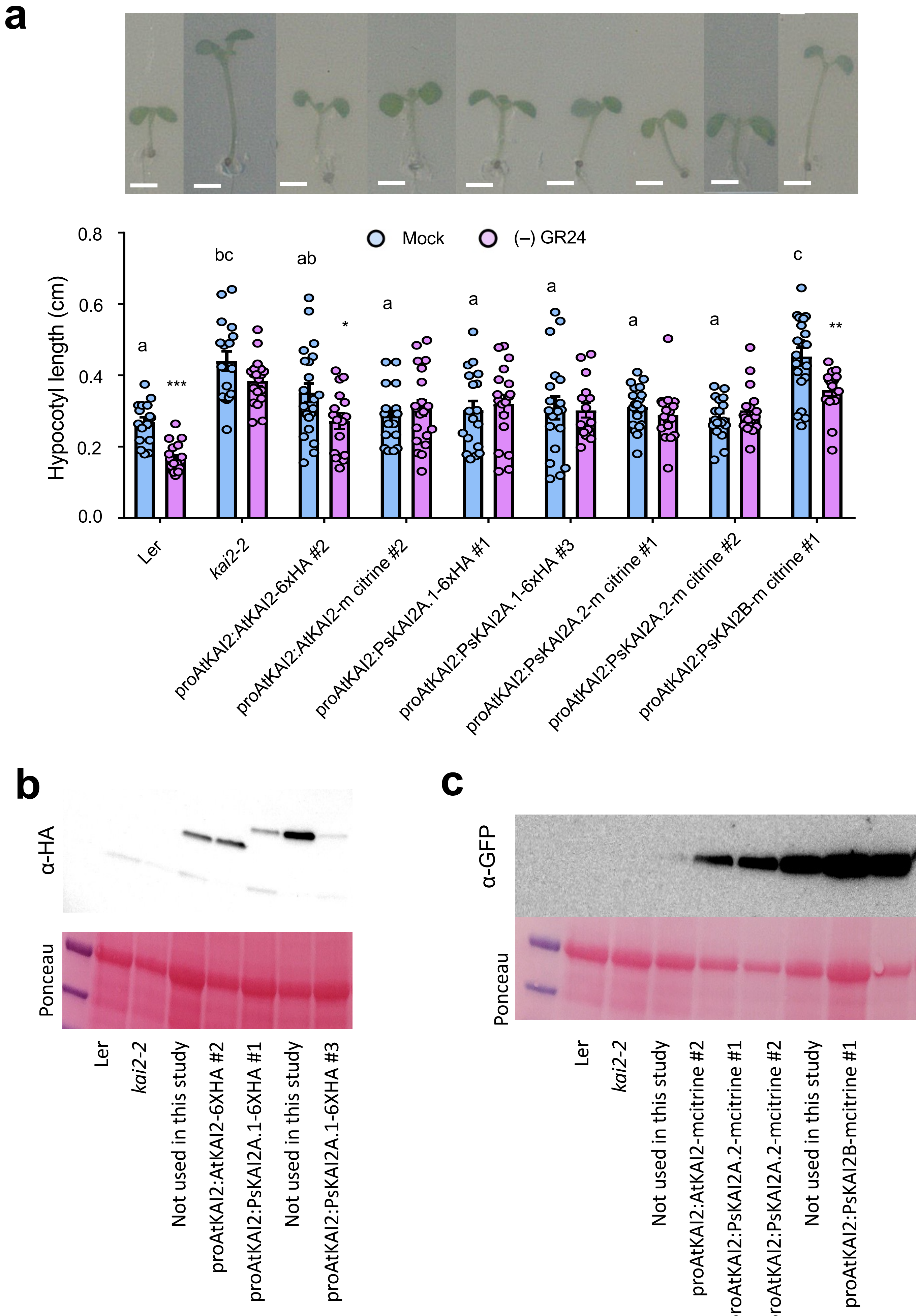
a
Hypocotyl length (cm)
0.0
0.2
0.4
0.6
0.8
Mock
(–) GR24
a
***
bc
ab
*
a
a
a
a
a
c
**
Ler
kai2-2
proAtKAI2:AtKAI2-6xHA #2
proAtKAI2:AtKAI2-m citrine #2
proAtKAI2:PsKAI2A.1-6xHA #1
proAtKAI2:PsKAI2A.1-6xHA #3
proAtKAI2:PsKAI2A.2-m citrine #1
proAtKAI2:PsKAI2A.2-m citrine #2
proAtKAI2:PsKAI2B-m citrine #1
b
α-HA
Ponceau
Ler
kai2-2
Not used in this study
proAtKAI2:AtKAI2-6XHA #2
proAtKAI2:PsKAI2A.1-6XHA #1
Not used in this study
proAtKAI2:PsKAI2A.1-6XHA #3
c
α-GFP
Ponceau
Ler
kai2-2
Not used in this study
proAtKAI2:AtKAI2-mcitrine #2
proAtKAI2:PsKAI2A.2-mcitrine #1
proAtKAI2:PsKAI2A.2-mcitrine #2
Not used in this study
proAtKAI2:PsKAI2B-mcitrine #1

Supplementary Fig. 6. Hypocotyl elongation in Ler *kai2-2* mutant and KAI2s protein expression in complementation assay. **(a)** Hypocotyl length of 7-day-old seedlings grown under low light at 21 °C. Data are means ± SE (n = 20-24; 2 plates of 10-12 seedlings per plate). Light blue bars: Mock (DMSO), lavender bars: (–)-GR24 (1µM). Complementation assays using the *AtKAI2* promoter to express *AtKAI2* (control) or *PsKAI2* genes in the null *kai2-2* mutant background (Ler ecotype) as noted above the graph. Proteins were tagged with 6xHA epitope or mCitrine protein. For DMSO controls, statistical differences were determined using a one-way ANOVA with a Tukey multiple comparison of means post-hoc test, statistical differences of $P<0.05$ are represented by different letters. Means with asterisks indicate significant inhibition compared to mock-treated seedlings with *** corresponding to $p \leq 0.001$ and * to $p \leq 0.01$, as measured by t- test. Picture are representative of the mock traiteent. Bar, 2 mm **(b-c)** AtKAI2 and PsKAI2 level analyzed by immunoblot using α-HA antibody **(b)** or α-GFP antibody **(c)** in wild type, *kai2-2* and transformed *kai2-2* plants, expressed under the control of *AtKAI2* promoter. Protein extracts from 10-d-old seedlings were separated by 10% SDS-PAGE. Ponceau staining is included for loading reference.

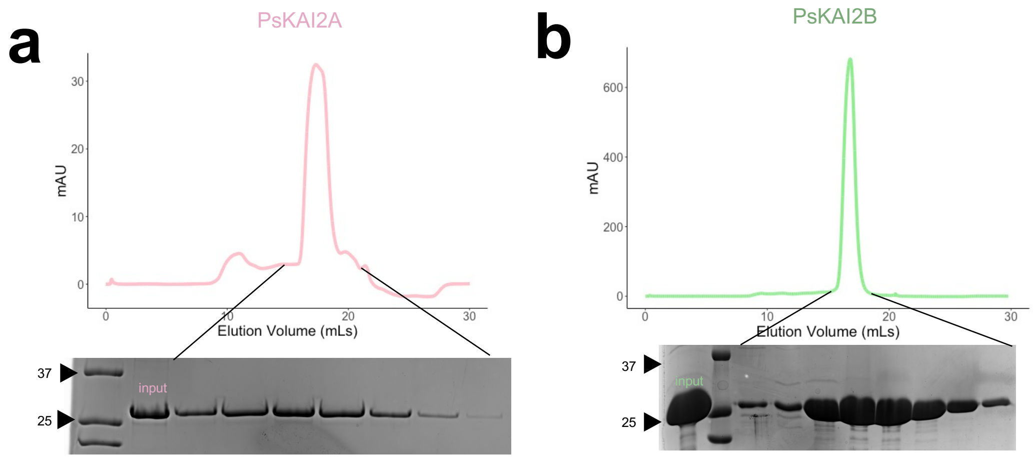

Supplementary Fig. 7. Purification of PsKAI2 Proteins. Size exclusion peaks eluates for PsKAI2A (**a**) and PsKAI2B (**b**). Proteins were resolved on SDS-PAGE gels and visualized via Coomassie stain. Molecular weight (MW) markers are labeled to show the relative size of proteins.

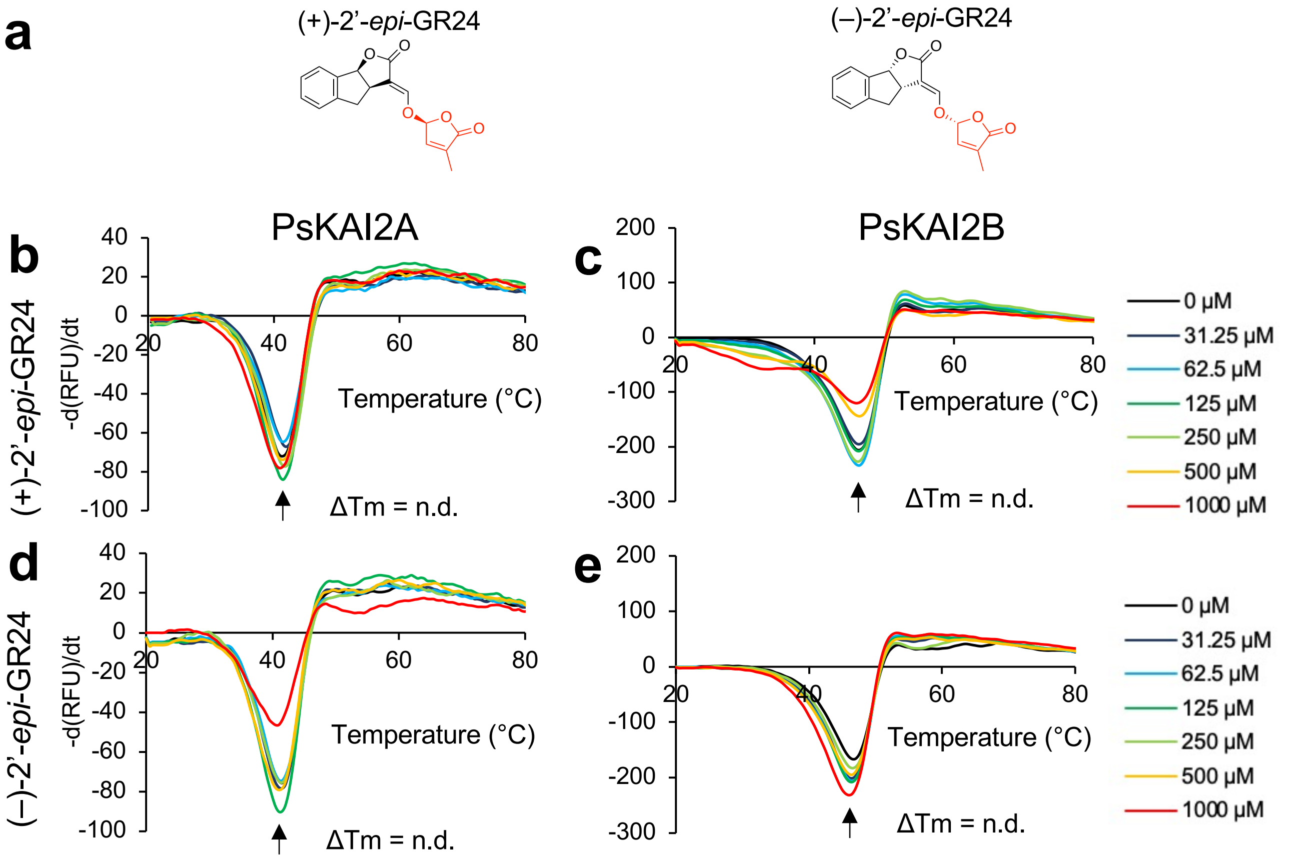

Supplementary Fig. 8. Biochemical analysis of the interaction between PsKAI2 proteins and the (+)-2’-*epi*-GR24 and (-)-2’-*epi*-GR24 isomers ligands by DSF. (**a**) Chemical structure of ligands used in DSF assay. The melting temperature curves of 10 μM PsKAI2A (**b**, **d**) or PsKAI2B (**c**, **e**) with (+)-*2’-epi*-GR24 (b-c), or (–)-*2’-epi*-GR24 (**d-e**) at varying concentrations are shown as assessed by DSF. Each line represents the average protein melt curve for three technical replicates and the experiment was carried out twice.

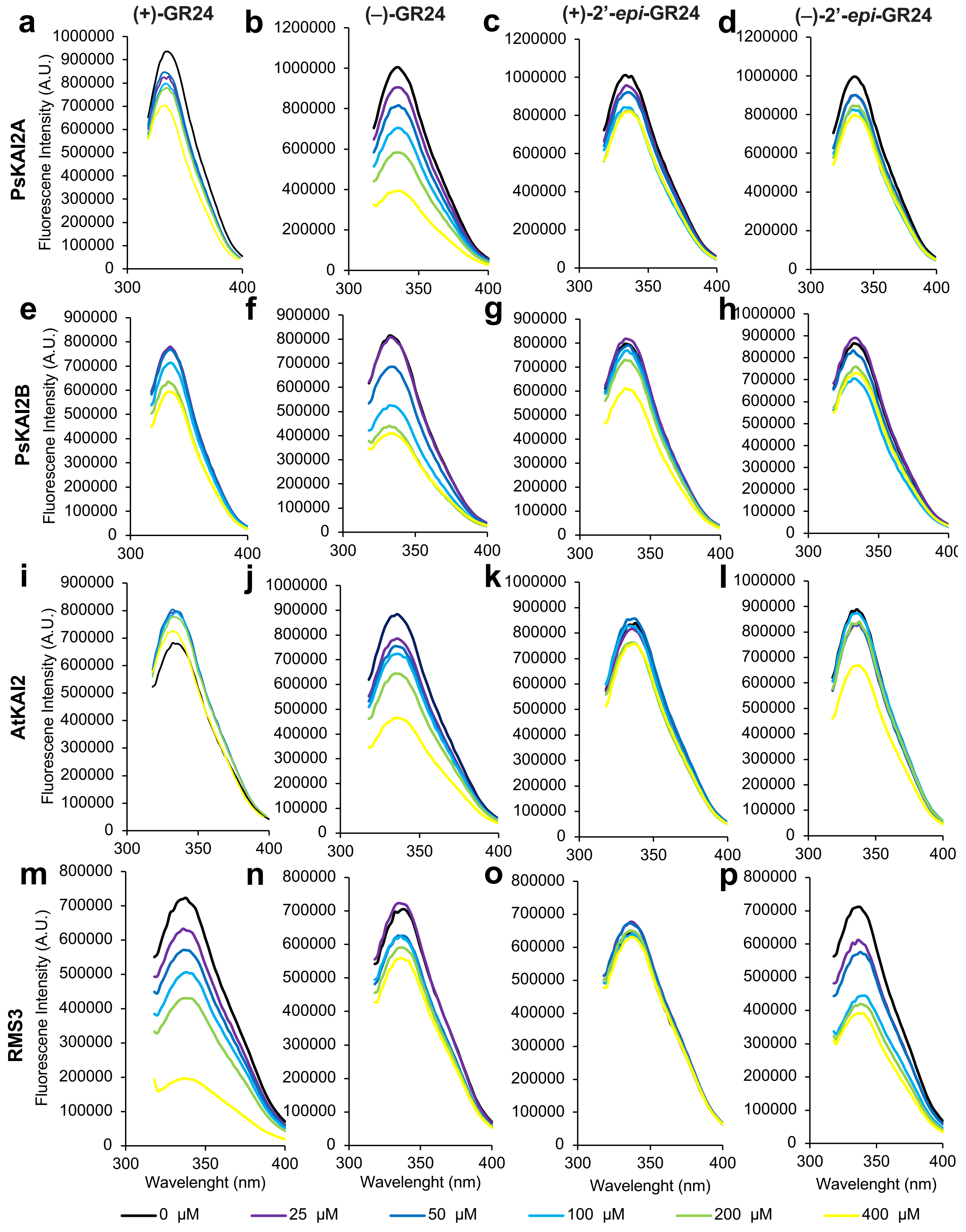
(+)-GR24
(–)-GR24
(+)-2’-epi-GR24
(–)-2’-epi-GR24
a
b
c
d
e
f
g
h
i
j
k
l
m
n
o
p
PsKAI2A
PsKAI2B
AtKAI2
RMS3
Fluorescene Intensity (A.U.)
Wavelenght (nm)
0 µM
25 µM
50 µM
100 µM
200 µM
400 µM

Supplementary Fig. 9. Intrinsic tryptophan fluorescence of PsKAI2s, AtKAI2 and RMS3 proteins in the presence of SL analogs. Intrinsic tryptophan fluorescence of PsKAI2A (**a-d**), PsKAI2B (**e-h**), AtKAI2 **(i-l)** and RMS3 (**m-p**) proteins in the presence of SL analogs. Changes in intrinsic fluorescence emission spectra in the presence of various concentrations of (+)-GR24 (**a;e;i;m;q**), (–)-GR24 (**b;f;j;n;r**), (+)-2'-*epi*-GR24 (**c;g;k;o;s**), (–)-2'-*epi*-GR24 (**d;h;l;p;t**). Proteins (10 µM) were incubated with increasing amounts of ligand (0–400 µM, top line to bottom line, respectively). The observed relative changes in intrinsic fluorescence were plotted as a function of SL analog concentration and transformed to degree of saturation and used to determine the apparent *K*D values relevant to **Fig. 4b**. The plots represent the mean of two replicates and the experiments were repeated at least three times. The analysis was performed with GraphPad Prism 8.0 Software.

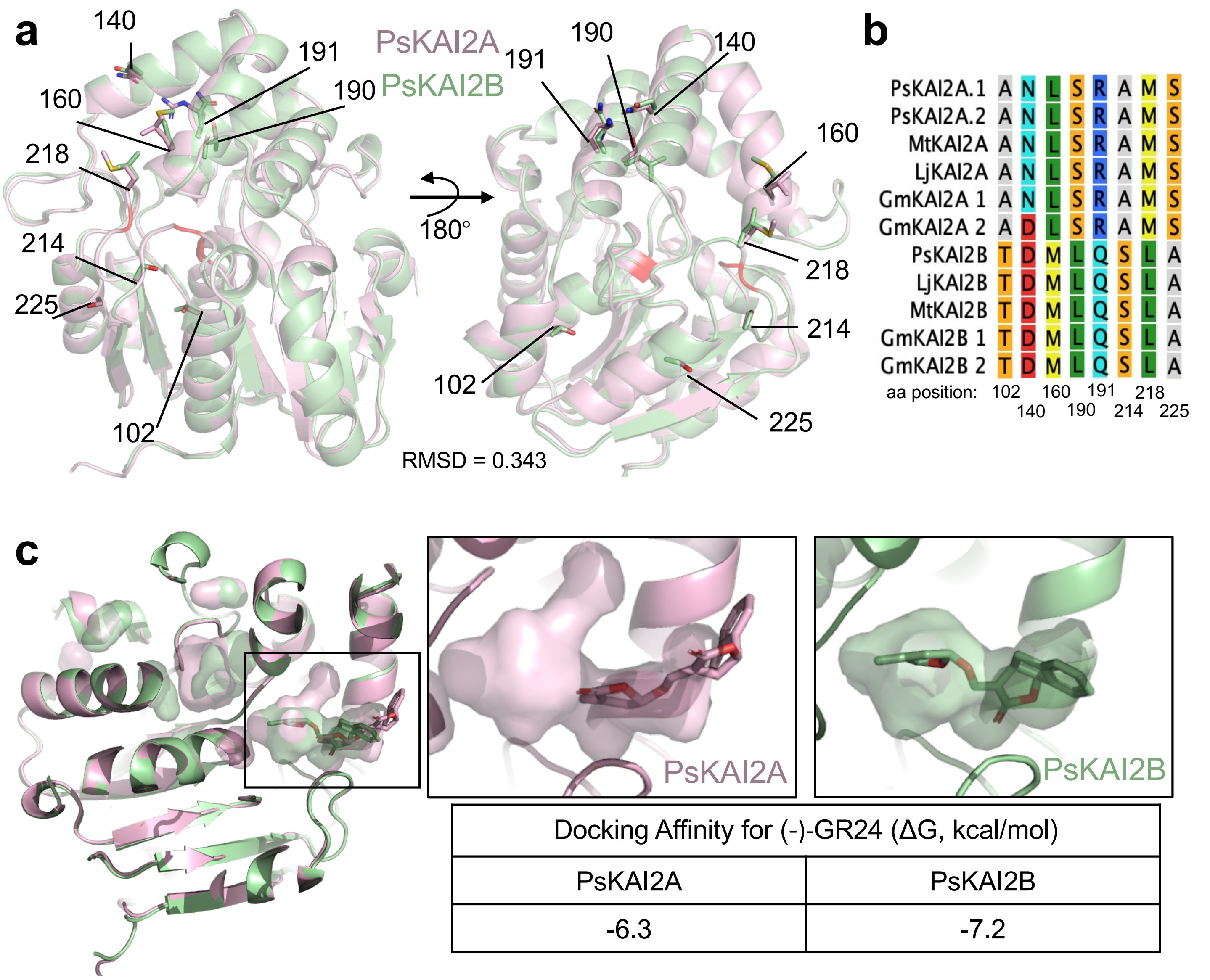

Supplementary Fig. 10. Structural divergence analysis of legume KAI2A and KAI2B and effect on ligand docking. (a) Structural alignment of PsKAI2A and PsKAI2B shown in pink and light green respectively. Calculated RMSD of aligned structures is shown. Residues differentiating all legume KAI2A from KAI2B are shown on each structure as sticks and labeled with residue number. Catalytic triad is shown in red. **(b)** Residues 102, 140, 160, 190, 191, 214, 218, and 225, L190, and L218 are highlighted as divergent legume KAI2 residues, conserved among all legume KAI2A or KAI2B sequences (with the exception of D140 in GmKAI2A 2) as shown in reduced Multiple Sequence Alignment from **Supplementary Fig. 1**. **(c)** *In silico* docking analysis of intact (–)-GR24 with solvent-accessible pocket shown for each structure and corresponding docking scores reported in kcal/mol.

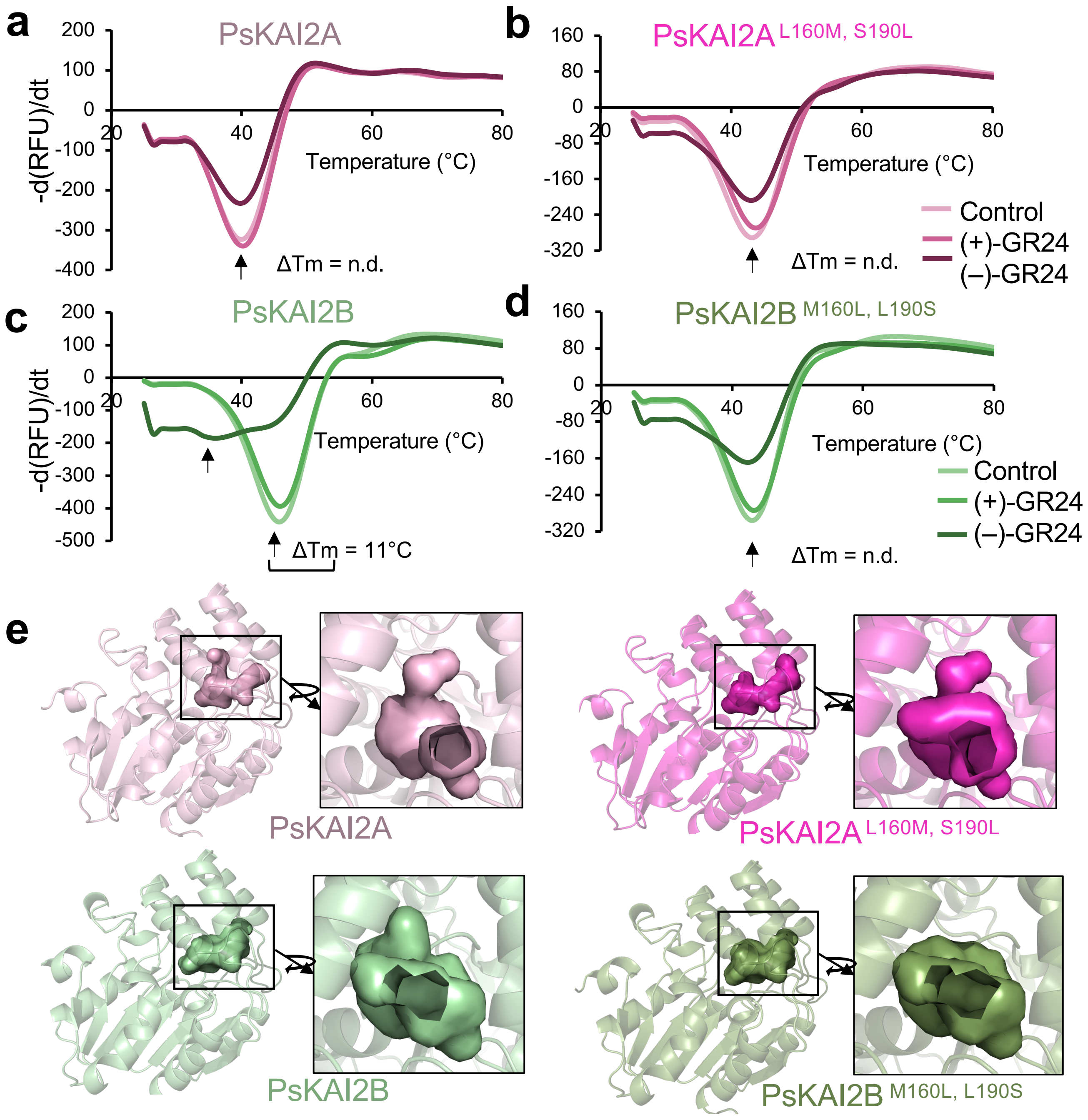

f

	SA vol.	SA area
PsKAI2A	114.8	231.2
PsKAI2B	125.4	234.4
PsKAI2A L160M, S190L	154.1	152.1
PsKAI2B M160\L, L190S	114.4	138.7

g

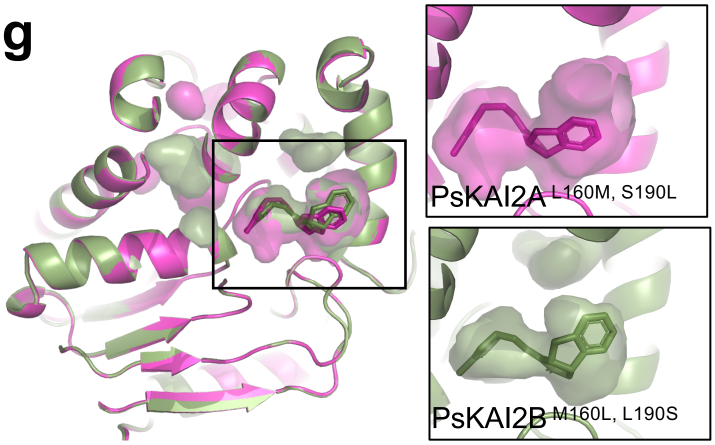

Docking Affinity for (-)-GR24 (ΔG, kcal/mol)	
PsKAI2A L160M, S190L	PsKAI2B M160\L, L190S
-1.7	-2.9

Supplementary Fig. 11. Biochemical and structural analysis of the interaction between wildtype and residue 160 and 190 swap mutant PsKAI2 proteins and (–)-GR24 by DSF and effect on ligand docking. The melting temperature curves of 10 μM PsKAI2A **(a)**, PsKAI2A $^{L160M,\ S190L}$ swap **(b)**, PsKAI2B **(c)**, and PsKAI2B $^{M160L,\ L190S}$ swap **(d)**, with (+)-GR24 or (–)-GR24 at the effective concentration of 62.5 μM are shown as assessed by DSF**.** Each line represents the average protein melt curve for three technical replicates and the experiment was carried out twice. **(e)** pocket surface representation of PsKAI2A model, PsKAI2B apo structure, and modelled PsKAI2A $^{L160M,\ S190L}$ and PsKAI2B $^{M160L,\ L190S}$ swap mutants. **(f)** Analysis of solvent accessible pocket volumes and areas calculated via the CASTp server. **(g)** **I**n silico docking analysis of intact (–)-GR24 with solvent-accessible pocket shown for each structure and corresponding docking scores reported in kcal/mol.

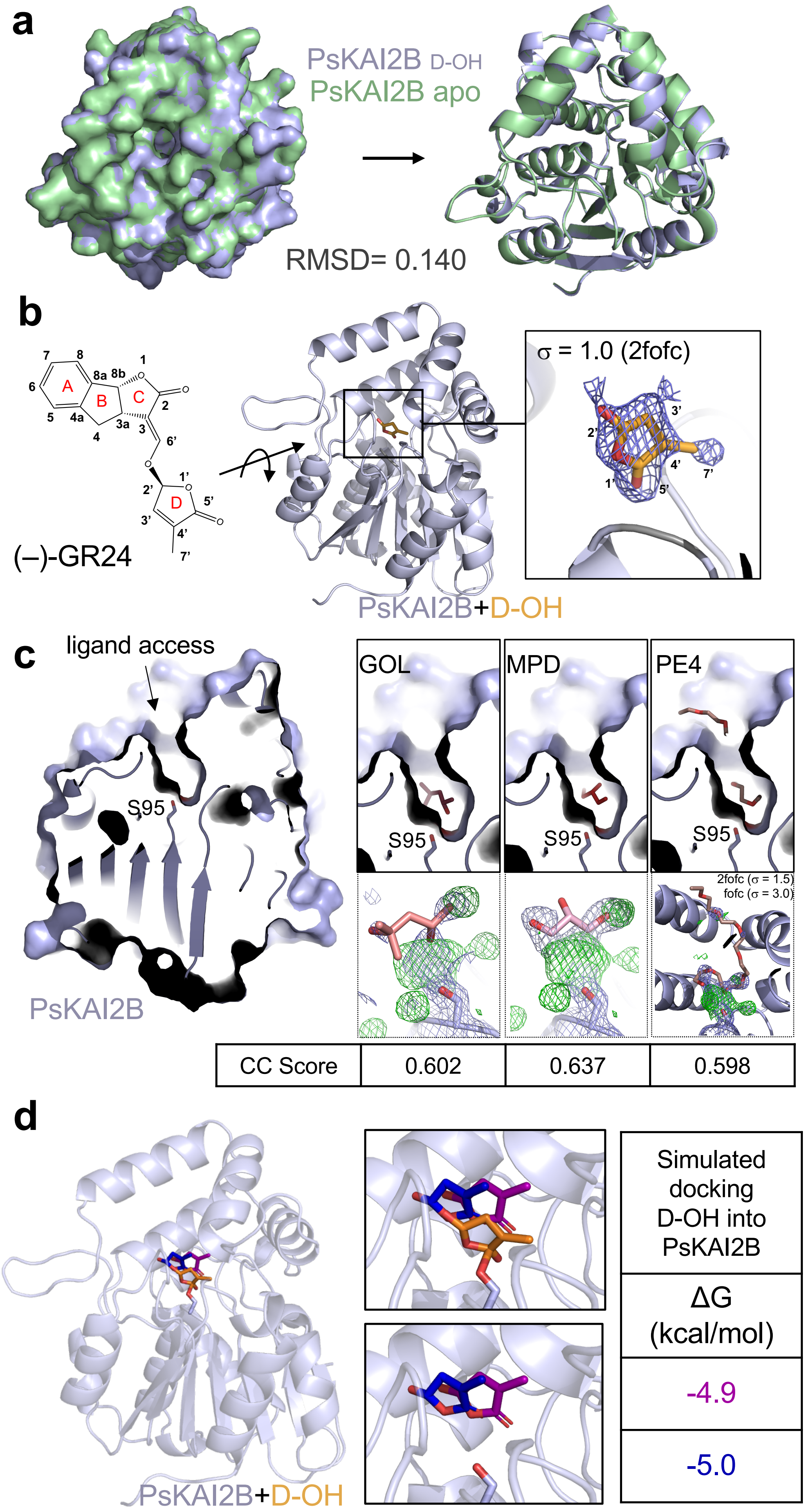

a
PsKAI2B D-OH
PsKAI2B apo
RMSD= 0.140
b
(–)-GR24
PsKAI2B+D-OH
σ = 1.0 (2fofc)
c
ligand access
S95
PsKAI2B
GOL
MPD
PE4
2fofc (σ = 1.5)
fofc (σ = 3.0)
CC Score
0.602
0.637
0.598
d
PsKAI2B+D-OH
Simulated docking D-OH into PsKAI2B
ΔG (kcal/mol)
-4.9
-5.0

Supplementary Fig. 12. Structural interrogation of the ligand bound PsKAI2B crystal structure. (a) Structural alignment of PsKAI2B apo (light green) and PsKAI2B in complex with (–)-GR24 D- OH (gray/blue). Calculated RMSD value is shown. Similar orientation of the superposition is shown in surface (right) and cartoon (left) representations. **(b)** Chemical structure of intact (–)- GR24 molecule with numbered carbons. Electron density mesh fit with D-OH ring of (–)-GR24. Protein structure is shown in blue/gray and ligand in orange. The electron density of the ligand is derived from 2mFoDFc (2fofc) map contoured at 1.0s. **(c)** LigandFit examination of crystallization and purification conditions reported here. Glycerol denoted: GOL, (+/-)-2-Methyl- 2,4-pentanediol denoted: MPD. Polyethylene glycol, PEG, denoted: PE4. Ligand access pocket is shown together with the ligand placed via LigandFit fitting software (top), and the obtained electron density maps following 3 cycles of Phenix refine. The electron densities shown are derived from 2mFoDFc map (2fofc, blue mesh) contoured at 1.5s and mFoDFc map (fofc, green mesh) contoured at 3s. Correlation Coefficient (CC) scores were calculated via LigandFit. **(d)** *In silico* analysis of D-OH ligand docking is shown in orange (found in the structure) and predicted orientations in navy and magenta with corresponding docking scores reported in kcal/mol.

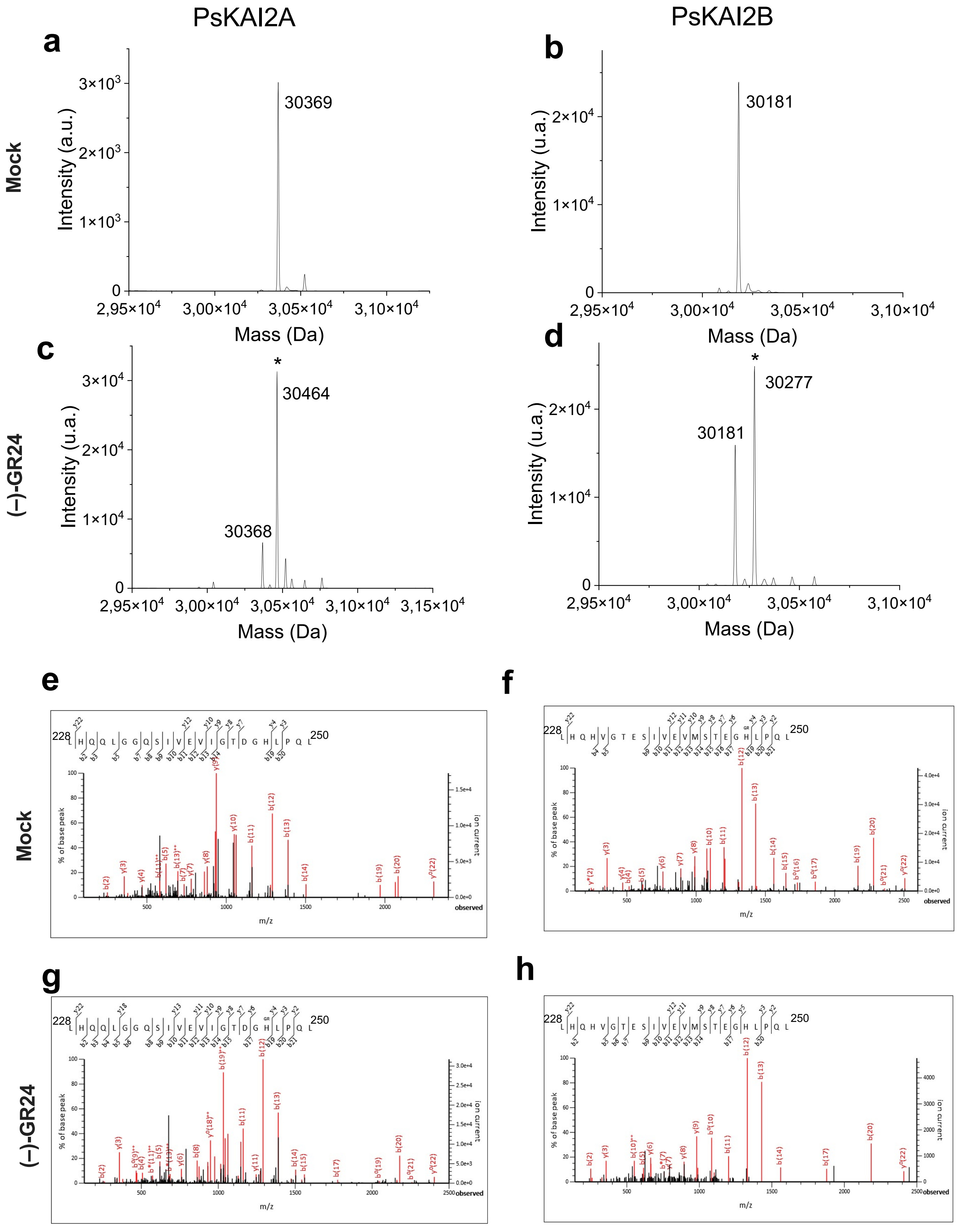

Supplementary Fig. 13. Mass spectrometry characterization of covalent PsKAI2-ligand complexes. A Deconvoluted electrospray mass spectra of PsKAI2A and PsKAI2B before (**a-b**) and after (**c-d**). adding of ligand (–)-GR24 are shown respectively on upper and lower panels. Peaks with an asterisk correspond to PsKAI2 covalently bound to a (–)-GR24 ligand (PsKAI2-ligand). A mass increment of 96 Da is measured for two PsKAI2-ligand complexes. (**e-h**) Ligand-modified H246 amino-acids were identified by nano LC-MSMS analyses after chymotrypsin proteolysis. Fragmentation spectra of unmodified and ligand-modified peptides are shown. Labeled peaks correspond to b and y fragments of the triple charged precursor ion. Histidine 246 residue modified by ligand is marked with GR on the sequence displayed on the top.

Supplementary Table 1. List of the mutations identified during TILLING and mutant alleles used in the study.

***PsKAI2A* TILLING mutants**				
**Mutant allele**	**Base position[1]**	**Protein position**	**Type of mutation**	**Protein location of the mutated amino acid**
Pskai2a-1	G266A	C89Y	Missense	
2	C333T	L111L	Silent	
Pskai2a-2	G368A	R123K	Missense	This R is conserved across KAI2s, located at the left base of the V lid in the back of a loop
4	G368A	R123K	Missense	
Pskai2a-3	G379A	D127N	Missense	This D is conserved across KAI2s, located at the left base of the V lid in the middle of a loop
6	C390T	Y130Y	Silent	
Pskai2a-4	G395A	G132E	Missense	This G is conserved across KAI2s, located on the same left loop region as *Pskai2*a-2 and *Pskai2a-3*, but are pointing more inwards so possibly closer to the entrance of the pocket
Pskai2a-5	G398A	G133E	Missense	see *Pskai2a-4*
9	G409A	E137K	Missense	
10	G412A	D138N	Missense	
11	G417A	L139L	Silent	
12	G417A	L139L	Silent	
13	G454A	A152T	Missense	
14	C465T	Y155Y	Silent	
15	G467A	G156E	Missense	
Pskai2a-6	G487A	G163R	Missense	This G is conserved across KAI2s, located at the right base of the V lid in a loop region, also pointing slightly inwards
17	G561A	L187L	Silent	
18	G676A	E226K	Missense	
19	G676A	E226K	Missense	
20	G715A	E239K	Missense	

***PsKAI2B* TILLING mutants**				
**Mutant allele**	**Base position[1]**	**Protein position**	**Type of mutation**	**Protein location of the mutated amino acid**
1	Before ATG	-		
2	44	T15I	Missense	
Pskai2b-1	34	V12I	Missense	This V is conserved across all KAI2s, located on the back of the protein pointing outwards not near lid or known binding interfaces
Pskai2b-2	254	R85K	Missense	This R is not conserved, AtKAI2 has a K at this position, located on the back of the protein pointing outwards not near lid or known binding interfaces
5	120	L40L	Silent	
6	121	L41L	Silent	
7	201	Y67Y	Silent	
Pskai2b-3	488	G163E	Missense	see *Pskai2a-6*
9	508	A170T	Missense	
10	655	A219T	Missense	
11	658	V220I	Missense	
12	664	V222I	Missense	
13	674	A225V	Missense	
14	540	N180N	Silent	
15	558	A186A	Silent	
16	663	P221P	Silent	

[1] base position from the ATG

Supplementary Table 2. List of primers used in this study

GENE	Primer name	Sequence	Observation
Primers for qRT-PCR analysis			
PsACTIN	PsACTIN_F1	GTGTCTGGATTGGAGGAT	Used in figure 1
	PsACTIN_R1	GGCCACGCTCATCATATT	
PsTUB	ST206-F	CAGAACAAGAACTCGTCATACT	Used in figure 2
	ST207-R	AGCCTTCCTCCTGAACATA	
PsKAI2B Psat4g083040	PsKAI2B_F1	GCCCTAAGCGTGTTACAAAC	Used in figure 2
	PsKAI2B_R1	ACTCGGTGCCGACGTGT	
	PsKAI2B_F2	GCCCTAAGCGTGTTACAAAC	Used in figure 1
	PsKAI2B_R2	ACTCGGTGCCGACGTGT	
PsKAI2A Psat2g169960	PsKAI2A_F1	CTTTGATAGTGTCGAGGACGA	Used in figure 2
	PsKAI2A_R1	GACCACCCAATTGTTGATGT	
	PsKAI2A_F2	CTTTGATAGTGTCGAGGACGA	Used in figure 1
	PsKAI2A_R2	GACCACCCAATTGTTGATGT	
PsDLK2 Psat1g039520	ST208-F	AGGCTTGTTCTTCTTGGTGC	Used in figure 2
	ST209-R	ATCTGAACTTGCAAATCCTCCC	
Primers for TILLING			
PsKAI2A Psat2g169960	PsKAI2A_N1F	GGAAACACTTTGTACCACATCTCG	Nested 1 PCR
	PsKAI2A_N1R	TCCTTCATCTTGTGCCTTACTAGC	
	PsKAI2A_N2Ftag	ACGACGTTGTAAAACGACGATAACATGGGTGCTGG	Nested 2 PCR Specific primers (black nucleotides) have a M13 tag (red nucleotide) in 5'end.
	PsKAI2A_N2Ftag	TAACAATTTCACACAGGCAATGGGCCTATAATGGG	
PsKAI2B Psat4g083040	PsKAI2B_Fw1	TTCCCTACACGACGCTCTTCCGATCTCAGCCTACAG TTATCAACAAACC	Specific primers (black nucleotides) have a Illumina adaptator (red nucleotide) in 5'end.
	PsKAI2B_Rv1	AGTTCAGACGTGTGCTCTTCCGATCTATTTTGGCAA ACAAATCAGG	
	PsKAI2B_Fw2	TTCCCTACACGACGCTCTTCCGATCTCGACAGTAAT TACTTTGGAGG	
	PsKAI2B_Rv2	AGTTCAGACGTGTGCTCTTCCGATCTTCACCGGAAT AACAACATCC	
Primers for splicing variant identification			
PsKAI2A Psat2g169960	*PsKAI2A*_F100	AACTCACACAGTCGGTGAAT	
	*PsKAI2A*_F898	ACCCCACAATATATAGTTGTTG	
	*PsKAI2A*_R1233	ATGCCACTTCCTTCATCTTG	
Primers for PsKAI2C detection			
PsKAI2C Psat3g014200	PsKAI2C-F171	CCCTGACTACTTTGGTTTTCAGT	
	PsKAI2C-R799	CAAGTTGAATATGTTTAAACCA	
PsEF1α Psat1g082600	PsEF1α_F2	GTACTCCAAGGGCAGGTATGAG	
	PsEF1α_R	CACTGGCACAGTTCCAATACC	
Primers for protein preparation and purification			
PsKAI2A Psat2g169960	PsKAI2A_F	aaaacctctacttccaatcgATGGGGATAGTGGAAGAAG	
	PsKAI2A.1_R	ccacactcatcctccggTTACAAATCTGCCTCAAGTTTC	
	PsKAI2A.2_R	ccacactcatcctccggTTACCTTATTGGCTCAATATTAAGT TG	
PsKAI2B Psat4g083040	PsKAI2B_F	aaaacctctacttccaatcgATGGGAATAGTGGAAGAAGC	
	PsKAI2B_R	ccacactcatcctccggTCAAGCTACAATATCATAACGAAT G	
Primers for generation of Arabidopsis transgenic lines			
PsKAI2A Psat2g169960	PsKAI2A_attb1	GGGGACAAGTTTGTACAAAAAAGCAGGCTtcATGGG GATAGTGGAAGAAGCA	
	PsKAI2A.1_attb2	ggggaccactttgtacaagaaagctgggtcCAAATCTGCCTCAA GTTTCA	
	PsKAI2A.2_attb2	ggggaccactttgtacaagaaagctgggtcCCTTATTGGCTCAAT ATTAA	
PsKAI2B Psat4g083040	PsKAI2B_attb1	GGGGACAAGTTTGTACAAAAAAGCAGGCTtcATGGG AATAGTGGAAGAAGC	
	PsKAI2B_attb2	ggggaccactttgtacaagaaagctgggtcAGCTACAATATCATA ACGAA	
AtKAI2 At4g37470	AtKAI2_attb1	ggggacaagtttgtacaaaaaagcaggcttcATGGGTGTGGTAG AAGAAGC	
	AtKAI2_attb2	ggggacaagtttgtacaaaaaagcaggcttcATGGGTGTGGTAG AAGAAGC	
