# Supplementary material for: Structural and functional analyses explain Pea KAI2 receptor diversity and reveal stereoselective catalysis during signal perception
Source: Commun Biol. 2022 Feb 11;5:126. doi: 10.1038/s42003-022-03085-6 (PMC8837635; doi:10.1038/s42003-022-03085-6)
Supplement: Supplementary file 2 — Description of Additional Supplementary Files [file 42003_2022_3085_MOESM2_ESM.pdf]

Description of Additional Supplementary Files

File name: Supplementary Data 1
Description: Source data for Fig. 1-6, 8, and Supplementary Fig. 5-6, 8-9, 11.
