# Supplementary material for: Structural and functional analyses explain Pea KAI2 receptor diversity and reveal stereoselective catalysis during signal perception
Source: Commun Biol. 2022 Feb 11;5:126. doi: 10.1038/s42003-022-03085-6 (PMC8837635; doi:10.1038/s42003-022-03085-6)
Supplement: Supplementary file 4 — Reporting Summary [file 42003_2022_3085_MOESM4_ESM.pdf]

nature portfolio

Corresponding author(s): Nitzan Shabek
nshabek@ucdavis.edu
Alexandre de SAINT GERMAIN
alexandre.de-saint-germain@inrae.fr

Last updated by author(s): Jan 8, 2022

Reporting Summary

Nature Portfolio wishes to improve the reproducibility of the work that we publish. This form provides structure for consistency and transparency in reporting. For further information on Nature Portfolio policies, see our Editorial Policies and the Editorial Policy Checklist.

Statistics

For all statistical analyses, confirm that the following items are present in the figure legend, table legend, main text, or Methods section.

n/a	Confirmed	
☐	☒	The exact sample size (*n*) for each experimental group/condition, given as a discrete number and unit of measurement
☐	☒	A statement on whether measurements were taken from distinct samples or whether the same sample was measured repeatedly
☐	☒	The statistical test(s) used AND whether they are one- or two-sided<br>*Only common tests should be described solely by name; describe more complex techniques in the Methods section.*
☒	☐	A description of all covariates tested
☐	☒	A description of any assumptions or corrections, such as tests of normality and adjustment for multiple comparisons
☐	☒	A full description of the statistical parameters including central tendency (e.g. means) or other basic estimates (e.g. regression coefficient) AND variation (e.g. standard deviation) or associated estimates of uncertainty (e.g. confidence intervals)
☐	☒	For null hypothesis testing, the test statistic (e.g. *F*, *t*, *r*) with confidence intervals, effect sizes, degrees of freedom and *P* value noted<br>*Give P values as exact values whenever suitable.*
☒	☐	For Bayesian analysis, information on the choice of priors and Markov chain Monte Carlo settings
☒	☐	For hierarchical and complex designs, identification of the appropriate level for tests and full reporting of outcomes
☒	☐	Estimates of effect sizes (e.g. Cohen's *d*, Pearson's *r*), indicating how they were calculated

Our web collection on statistics for biologists contains articles on many of the points above.

Software and code

Policy information about availability of computer code

Data collection	Single crystal X-ray diffraction data were collected at Advanced Light Source (ALS) in Berkeley. The obtained data were integrated and scaled with HKL2000. The structure model was manually built, refined, and rebuilt with PHENIX and COOT
Data analysis	Structure analysis were carried out using Coot, PyMOL, PHENIX. Kinetics analysis were performed using The analysis was performed with GraphPad Prism 7.05 Software.

For manuscripts utilizing custom algorithms or software that are central to the research but not yet described in published literature, software must be made available to editors and reviewers. We strongly encourage code deposition in a community repository (e.g. GitHub). See the Nature Portfolio guidelines for submitting code & software for further information.

Data

Policy information about availability of data

All manuscripts must include a data availability statement. This statement should provide the following information, where applicable:
- Accession codes, unique identifiers, or web links for publicly available datasets
- A description of any restrictions on data availability
- For clinical datasets or third party data, please ensure that the statement adheres to our policy

The atomic coordinates of KAI2 structures have been deposited in the Protein Data Bank with accession codes 7K2Z and 7K38

Field-specific reporting

Please select the one below that is the best fit for your research. If you are not sure, read the appropriate sections before making your selection.

☒ Life sciences ☐ Behavioural & social sciences ☐ Ecological, evolutionary & environmental sciences

For a reference copy of the document with all sections, see nature.com/documents/nr-reporting-summary-flat.pdf

Life sciences study design

All studies must disclose on these points even when the disclosure is negative.

Sample size	No sample-size calculations were performed; for Fig1-2, we used similar or higher sample sizes as in our previous studies (i.e. de Saint germain et al 2016).
Data exclusions	No data were excluded
Replication	Each experiment was reproduced at least three times on separate occasions. Experimental findings were reliably reproduced.
Randomization	Animal experiments were not performed in this study, so no randomization was needed.
Blinding	Animal experiments were not performed in this study, so Investigators were not blinded to the experiment

Reporting for specific materials, systems and methods

We require information from authors about some types of materials, experimental systems and methods used in many studies. Here, indicate whether each material, system or method listed is relevant to your study. If you are not sure if a list item applies to your research, read the appropriate section before selecting a response.

Materials & experimental systems

n/a	Involved in the study
☒	☐ Antibodies
☒	☐ Eukaryotic cell lines
☒	☐ Palaeontology and archaeology
☒	☐ Animals and other organisms
☒	☐ Human research participants
☒	☐ Clinical data
☒	☐ Dual use research of concern

Methods

n/a	Involved in the study
☒	☐ ChIP-seq
☒	☐ Flow cytometry
☒	☐ MRI-based neuroimaging
